# Comparison of Alternative Evidence Summary and Presentation Formats in Clinical Guideline Development: A Mixed-Method Study

**DOI:** 10.1371/journal.pone.0055067

**Published:** 2013-01-25

**Authors:** Newton Opiyo, Sasha Shepperd, Nyokabi Musila, Elizabeth Allen, Rachel Nyamai, Atle Fretheim, Mike English

**Affiliations:** 1 Health Services Research Group, KEMRI-Wellcome Trust Research Programme, Nairobi, Kenya; 2 Institute of Health and Society, University of Oslo, Oslo, Norway; 3 Department of Public Health, University of Oxford, Oxford, United Kingdom; 4 Department of Medical Statistics, London School of Hygiene and Tropical Medicine, London, United Kingdom; 5 Division of Paediatrics, Ministry of Medical Services, Nairobi, Kenya; 6 Global Health Unit, Norwegian Knowledge Centre for Health Services, Oslo, Norway; 7 Department of Paediatrics, University of Oxford, Oxford, United Kingdom; Tehran University of Medical Sciences, Islamic Republic of Iran

## Abstract

**Background:**

Best formats for summarising and presenting evidence for use in clinical guideline development remain less well defined. We aimed to assess the effectiveness of different evidence summary formats to address this gap.

**Methods:**

Healthcare professionals attending a one-week Kenyan, national guideline development workshop were randomly allocated to receive evidence packaged in three different formats: systematic reviews (SRs) alone, systematic reviews with summary-of-findings tables, and ‘graded-entry’ formats (a ‘front-end’ summary and a contextually framed narrative report plus the SR). The influence of format on the proportion of correct responses to key clinical questions, the primary outcome, was assessed using a written test. The secondary outcome was a composite endpoint, measured on a 5-point scale, of the clarity of presentation and ease of locating the quality of evidence for critical neonatal outcomes. Interviews conducted within two months following completion of trial data collection explored panel members’ views on the evidence summary formats and experiences with appraisal and use of research information.

**Results:**

65 (93%) of 70 participants completed questions on the prespecified outcome measures. There were no differences between groups in the odds of correct responses to key clinical questions. ‘Graded-entry’ formats were associated with a higher mean composite score for clarity and accessibility of information about the quality of evidence for critical neonatal outcomes compared to systematic reviews alone (adjusted mean difference 0.52, 95% CI 0.06 to 0.99). There was no difference in the mean composite score between SR with SoF tables and SR alone. Findings from interviews with 16 panelists indicated that short narrative evidence reports were preferred for the improved clarity of information presentation and ease of use.

**Conclusions:**

Our findings suggest that ‘graded-entry’ evidence summary formats may improve clarity and accessibility of research evidence in clinical guideline development.

**Trial Registration:**

Controlled-Trials.com ISRCTN05154264

## Introduction

Policy decisions about which interventions to implement, modify or withdraw from health care, should be informed by the best available evidence. While systematic reviews are a key source of evidence [1], [2], technical language and the time it takes to read and identify the key findings in a review, may deter healthcare decision makers from applying this evidence [3]. Thus, alternative ways of summarising and presenting evidence have been developed to improve accessibility and use [Box S1]. Such summaries need to be up to date and well aligned to the needs of policymakers and clinicians. Most examples and most evaluations of their effectiveness in supporting healthcare decision come from high-income countries (HICs) [4], [5]. Experience with existing research synthesis (systematic review derived) products [Box S1], and the required adaptations to formats for their effective use in low-income countries, remain under researched. In this paper we report the findings of a mixed-method evaluation of the usefulness of these products, compared with more traditional formats, for a large, multidisciplinary guideline development panel in Kenya.

## Methods

### Ethics Statement

Ethical approval for the conduct of this study was granted by the Kenya Medical Research Institute scientific committee and National Ethics Review Committee in Kenya (SSC Protocol No 1770). Individual written informed consent for participation and audio recording of discussions was obtained prior to the face-to-face interviews. Confidentiality of participant information was ensured by assigning anonymous codes to individual audio interviews and transcripts.

### Study Design

This was a mixed-method study incorporating a randomised controlled trial (RCT) to assess the effectiveness of three different evidence summary formats with semi-structured follow-up interviews to explore panel members’ views of these formats, experience with appraisal, use of and engagement with the research evidence.

### Study Setting

The Kenya Medical Research Institute - Wellcome Trust Research Programme together with Kenya’s Ministry of Medical Services collaborated to host a one-week national guideline development workshop (‘Child Health Evidence Week’) [6], held in Nairobi, Kenya, between 21st and 25th June, 2010.

### Participants

Trial participants consisted of a multidisciplinary panel of healthcare professionals with varied roles in neonatal and paediatric policy and care in Kenya (table 1). All 77 participants nominated by their departmental heads to participate in the one-week national guideline development workshop were eligible for recruitment. The Ministry of Medical Services sent invitation letters to those nominated, describing their expected roles (e.g. pre-reading of provided research evidence) and the research component of the workshop. We contacted those nominated by phone to confirm their agreement to participate and to further explain workshop procedures. We made follow-up telephone calls to non-responsive participants. Participants who declined participation at this stage were excluded from the sampling list; this reduced our sample size to 70 participants. For the interviews, we purposively selected a sub-sample of stakeholders (n = 16) who attended the guideline development workshop to: (1) achieve representation from groups involved at different levels of the health system; and (2) maximise the variety of views and experiences examined.

**Table 1:** Profile of guideline panel members.

Characteristic	Frequency	%		
**Sex**				
Male	34	49		
Female	36	51		
**Age (years)**				
21–30	8	11		
31–40	35	50		
41–50	14	20		
51–60	10	14		
Above 60	3	4		
**Profession**				
Paediatrician	32	46		
Medical Officer	11	16		
Nursing Officer	7	10		
Research (research supervision) role	5	7		
Trainer of healthcare workers	5	7		
National or provincial role for MoM/MoPHS	4	6		
Clinical Officer	3	4		
Pharmacist	2	3		
Academic administration	1	1		
**Number of years as a healthcare professional**				
3–7	23		33	
8–12	14		20	
13–21	16		23	
22–40	17		24	

### Interventions

We assembled evidence in three formats: systematic reviews (SR) alone (pack ‘A’), systematic reviews with summary-of-findings tables (SR with SoF tables; pack ‘B’) and ‘graded-entry’ formats (pack ‘C’). Evidence pack ‘A’ represented the common standard practice of using systematic reviews and lengthy technical reports to inform healthcare policy and guideline development. Evidence pack ‘B’ represented the recently enriched format for preparing full Cochrane reviews [5].

Evidence pack C was designed to allow stepwise access (i.e. ‘graded-entry’) to the evidence. It started with a ‘front-end’ short interpretation of the main findings and conclusions, drawn from evidence synthesis (webappendix S1). These front-end concise summaries were followed by a locally prepared, short, contextually framed, narrative report (hereafter called a narrative report) [7] in which the results of the systematic review (and other evidence where relevant) were described and locally relevant factors that could influence the implementation of evidence-based guideline recommendations (e.g. resource capacity) were highlighted. The front-end summary and the narrative report were combined with the full systematic review (e.g. as published by the Cochrane Collaboration) to make a three-component set branded pack ‘C’.

The ‘front-ends’ in pack ‘C’ were adapted from existing review-derived formats designed to allow rapid scanning for key messages [Box S1]. Further development of these formats was guided by theoretical frameworks on information transfer for non-research audiences [8]–[10]. Briefly, these frameworks propose that to improve knowledge transfer dissemination materials need to be easy to use, accessible, credible (trustworthy) and desirable. The content needs to be current, relevant, methodologically competent and comprehensive. Finally, the ‘messages’ need to be tailored to the specific needs and context of the users.

The GRADE (Grading of Recommendations Assessment, Development, and Evaluation) system [11] was used to appraise and summarise evidence in summary-of-findings tables. These tables were included in the front-end summaries and narrative reports. The summaries were delivered to participants as pre-reading materials one month before the workshop.

### Randomisation/Outcome Measures

**Figure 1:**
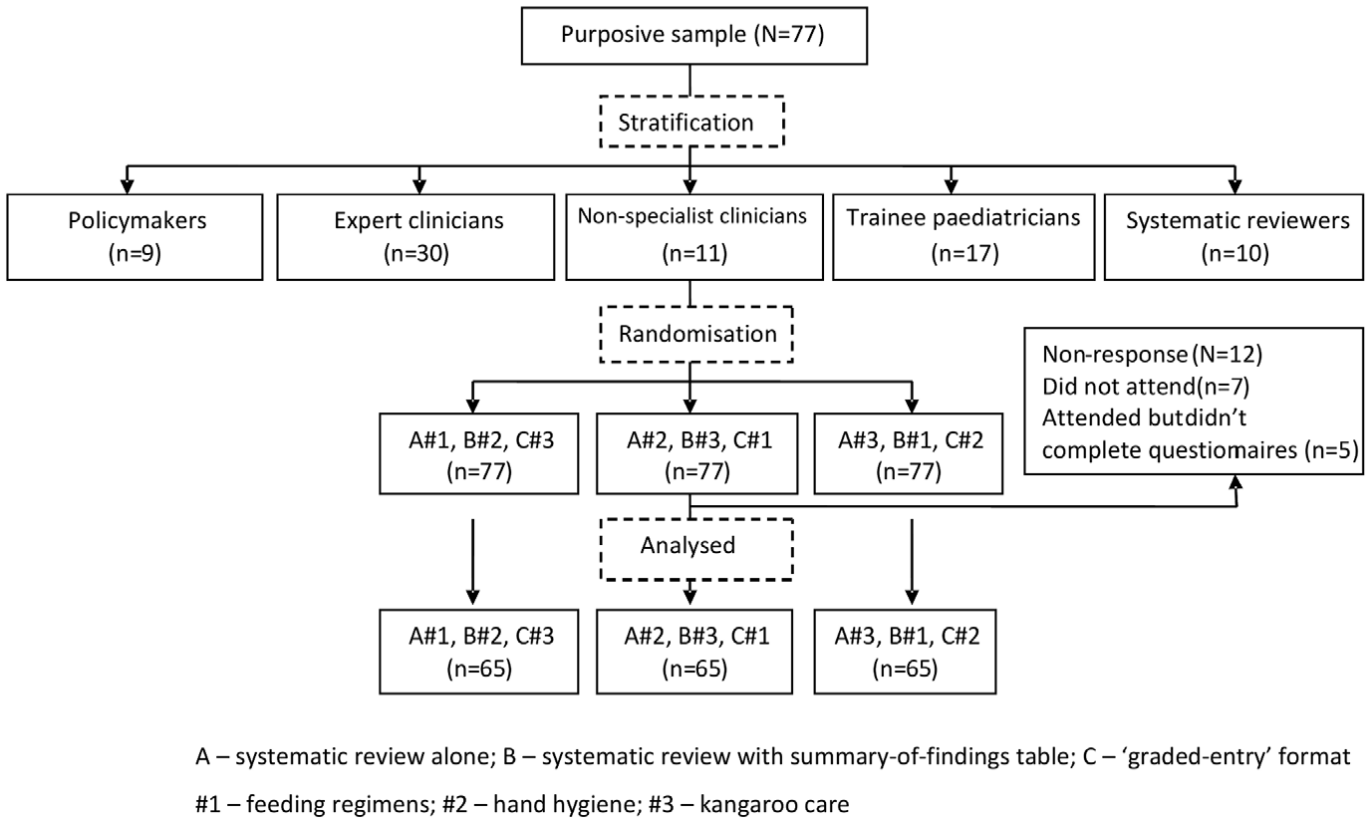
Trial profile.

Evidence summaries in pack A, B and C formats were prepared for three ‘tracer-interventions’ relevant to neonatal care where new guidelines were being considered and for which systematic reviews had been recently published: (1) feeding regimens in sick newborns [12]; (2) hand hygiene for infection prevention [13]; and (3) kangaroo care for low birth weight babies [14]. For example, for feeding regimens we packaged evidence in the format of a systematic review (pack ‘A’), a systematic review with summary-of-findings tables (pack ‘B’) and a ‘graded-entry’ format (pack ‘C’); a similar approach was used to assemble differing evidence for hand hygiene and kangaroo care. We then provided all individual participants with evidence on all three tracer topics but used randomisation, within 5 professional strata, to ensure that all participants received one tracer-topic with packaging approach A, one with packaging approach B and one with packaging approach C. This enabled us, when examining which package is most effective at conveying research information, to address the possibility that the relationship is confounded by the nature of the topic and, in subsequent interviews, explore participants’ views on all the 3 evidence packaging formats. Further details on the randomisation process is summarised in webappendix S2 and Figure 1.

The primary outcome was the proportion of correct responses to key clinical questions relevant to the specific tracer topics. This tested the understanding of the effects of tracer-interventions on critical neonatal outcomes (mortality, morbidity). The secondary outcome measure was a composite score representing participants’ self-reports of the clarity and accessibility of the evidence; participants rated their responses on a 3 or 5-point scale. These domains have successfully been tested and used in previous trials [5], [15], [16].

### Data Collection

Participants completed questionnaires on the first day (June 21st, 2010) of the guideline development workshop before the panel discussions about guidance recommendations. The questionnaires were adapted from existing studies [5], [15], [16], and required responses from participants on: (1) personal characteristics (e.g. age, sex); (2) experience with interpreting research evidence and evidence based medicine skills; (3) recognition and understanding of key messages; (4) ease of access to and perceived value of provided evidence summaries; and (5) preferred summary formats. The questionnaires were pilot-tested on two occasions on a multi-disciplinary group of 18 local researchers and clinicians and further refined based on feedback received. Participants were allowed up to 45 minutes to complete the questionnaire during which they had access to their personalized ‘evidence packs’.

Individual face-to-face interviews were conducted (between July and August 2010) by NO following completion of the questionnaire but before analysis. Interview questions were derived from a review of literature on best practices in clinical guideline development [17]–[19], pilot-tested (with three child health experts), and modified. The interviews lasted approximately 30 to 45 minutes and focused on collecting information about participants’ experiences with appraisal, the use of research evidence and views on evidence summary formats.

### Analysis

The sample size calculation was based on the pre-specified primary outcome assuming a two-way comparison of the three-component pack (C) versus the minimum standard pack (A) and a 1∶1 allocation ratio. The trial was designed to have 90% power at an alpha (significance level) of 0.05 to detect a 40% absolute difference in the proportion of correct responses with 35 observations on each evidence pack (calculated using a conventional two-sided Chi-squared test). Assuming a non-response rate of 10% we targeted at least 40 participants for randomisation. All analyses treated the respondent as a unit of clustering.

The crude odds (likelihood) of correct responses for pack C compared to the odds for pack A (assumed baseline pack) were estimated using logistic regression. The models were also adjusted for the effect of strata and the tracer intervention. Pack by strata interaction terms were included to assess whether the effect of the different evidence packs differed by strata. The secondary outcome measure was the clarity and accessibility score. The mean clarity and accessibility scores of pack C compared to the mean scores of pack A were estimated using linear regression and the lack of normality of the scores (webappendix S3) was accounted for using bootstrapping methods [20]. Similar processes were used to compare mean scores of pack B to A. To confirm the results of linear regression analysis, an alternative approach to the analysis was undertaken: the odds of a one point increase in the ‘clarity and accessibility’ scores of pack B and C compared to the odds of pack A were estimated using ordinal logistic regression models. Further tests of interactions between pack and strata were performed using likelihood ratio tests. Where evidence of interaction was found, stratum-specific ORs and 95% CIs were calculated. All analyses were done with STATA (version 11.0). Further details on data handling and analysis is summarised in webappendix S4.

Audio interviews were transcribed verbatim by NO. Emerging themes and concepts were extracted by at least two co-investigators (NO, SS, NM, AF) working independently [21]. These were compared and discussed; NO summarised the recurrent concepts into a set of initial descriptive themes with narrative summaries explaining each theme. These were discussed by investigators iteratively, with reference to the original interview transcripts, until a final set of themes was agreed upon.

Although quantitative and qualitative components of this study were pre-specified in the proposal integration occurred after separate analysis of each component at the point of interpretation of the results.

## Results

The trial profile is summarised in Figure 1. Seventy (91%) out of 77 participants invited attended the workshop. The most common reason for non-attendance was related to timing of the meeting. Table 1 shows the baseline characteristics of participants. A total of 16 participants were interviewed: three paediatricians from district hospitals, two senior paediatric trainees, one nurse manager, four trainers of healthcare workers (nursing and paediatric academics), one World Health Organization (WHO) officer, one research director and four government policymakers.

### Quantitative Findings

Sixty-five (93%) of 70 participants completed questions on primary outcome measures. A descriptive summary of the proportion of correct and incorrect responses for each evidence pack is shown in table 2. There were no significant differences between packs in the odds of correct responses (adjusted ORs: pack B versus A 0.59, 95% CI 0.32 to 1.07; pack C versus A 0.66, 95% CI 0.36 to 1.21; table 3). There was some evidence of differences in the odds of correct responses across different groups of healthcare professionals (p = 0.057). Results of sub-group analyses (although not statistically significant) suggested that both pack B (systematic reviews with SoF tables) and pack C (the three component, graded entry pack) improved understanding for policymakers (pack B: OR 1.5, 95% CI 0.15 to 15.15; pack C: OR 1.5, 95% CI 0.64 to 3.54) and trainee paediatricians (pack B: OR 1.3, 95% CI 0.37 to 4.66; pack C: OR 1.78, 95% CI 0.43 to 7.33).

**Table 2:** Proportion of incorrect and correct responses (N = 65 participants).

Responses	Pack A	Pack B	Pack C	Total
Incorrect	39 (30.0)	51 (39.2)	49 (38.0)	139 (35.7)
Correct	91 (70.0)	79 (60.8)	80 (62.0)	250 (62.3)
Total	130	130	129	389

Figures in brackets are percentages; pack A = systematic review; pack B = systematic review with summary-of-findings table; pack C = ‘graded-entry’ format (a ‘front-end’ summary of key information linked to a short contextually framed narrative report and full systematic review).

**Table 3:** Primary and secondary outcomes (N = 65 participants).

Odds ratios for correct responses					
Pack	Unadjusted OR (95% CI)		p-value	Adjusted OR (95% CI)†	p-value
Pack A	1 (Referent)		–	1	–
Pack B	0.66 (0.37–1.20)		0.366	0.59 (0.32–1.07)	0.220
Pack C	0.70 (0.39–1.27)			0.66 (0.36–1.21)	
**Mean differences in ‘value and accessibility’ scores**					
		**Mean difference (95% CI)**	**p-value**	**Mean difference (95% CI)** †	**p-value**
Pack A		1	–	1	–
Pack B		−0.14 (−0.77 to 0.49)	0.046	−0.11 (−0.71 to 0.48)	0.025
Pack C		0.49 (0.01 to 0.98)		0.52 (0.06 to 0.99)	
**Odds ratios for ‘value and accessibility’ scores**					
		**Unadjusted OR (95% CI)**	**p-value**	**Adjusted OR (95% CI)** †	**p-value**
Pack A		1	–	1	–
Pack B		0.97 (0.64–1.47)	0.038	0.91 (0.57–1.46)	0.022
Pack C		1.48 (1.06–2.08)		1.52 (1.06–2.20)	

OR = odds ratio; CI = confidence interval;

† Adjusted for type of strata and tracer-intervention; pack A =  systematic review; pack B =  systematic review with summary-of-findings table; pack C = ‘graded-entry’ format (a ‘front-end’ summary of key information linked to a short contextually framed narrative report and full systematic review); p-values - values for tests of significance of joint association between the summary formats and outcomes.

‘Graded-entry’ formats (pack C), compared to systematic review formats (pack A), were associated with a significantly higher mean ‘value and accessibility’ score (adjusted mean difference 0.52, 95% CI: 0.06 to 0.99; table 3). Similarly, pack C, compared to pack A, was associated with a 1.5 higher odds of judgments about the quality of evidence for critical neonatal outcomes being clear and accessible (adjusted OR: 1.52, 95% CI 1.06 to 2.20; table 3). There was no evidence that pack B (systematic reviews with SoF tables) improved this composite score compared to pack A (adjusted mean difference: −0.11, 95% CI −0.71 to 0.48; table 3).

More than half of the respondents (60%) found systematic reviews to be more difficult to read compared to narrative reports, but some (17%) responded that systematic reviews were easy to read. About half of the participants (51%) found systematic reviews to be easier to read compared to summary-of-findings tables (26%). A higher proportion of participants preferred evidence summarised in narrative report formats to the full version of the systematic reviews (53% versus 25%). See table 4 for a complete overview of these findings.

**Table 4:** Ease of use and summary format preferences.

	n/N†		
	Percentage (95% confidence interval)		
	Easy to read	Neutral	Difficult
How easy to read did you find evidence summarised in systematic reviewformats compared to narrative report formats	10/5817% (8%–27%)	13/5822% (12%–0.33%)	35/5860% (48%–73%)
How easy to read did you find evidence summarised in systematic reviewformats compared to summary-of-findings table formats	16/6126% (15%–37%)	14/6123% (13%–33%)	31/6151% (38%–63%)
	**Strongly prefer systematic review**	**Neutral**	**Strongly prefer narrative report**
Comparing systematic review versus narrative report formats	16/6425% (14%–36%)	14/6422% (12%–32%)	34/6453% (41%–65%)
	**Strongly prefer systematic review**	**Neutral**	**Strongly prefer SoF table**
Comparing systematic review versus summary-of-findings (SoF) table	20/6332% (20%–43%)	18/6329% (17%–40%)	25/6340% (27%–52%)
	**Strongly prefer narrative report**	**Neutral**	**Strongly prefer SoF table**
Comparing narrative report versus summary-of-findings (SoF) table	24/6438% (25%–50%)	25/6439% (27%–51%)	15/6423% (13%–34%)

† Denominators exclude missing data.

Participants’ self-rated experiences with research literature and familiarity with evidence based medicine terminology are presented in table 5. Of note are that about 29% of respondents had not read a systematic review in the last year. Most of the participants (53[82%]) responded that they spent less than 12 hours reading provided evidence summaries before the workshop and 6 (9%) reported not reading materials at all.

**Table 5:** Participants’ self-rated experience with research literature and familiarity with evidence-based medicine terminologies†.

Characteristic	Frequency (%)
**Frequency of reading journal articles in the last one year (n = 65)**	
Less than once per year	16 (24.6%)
1 to 4 times per year	15 (23.1%)
5 to 10 times per year	13 (20.0%)
More than 10 times per year	21 (32.3%)
**Frequency of reading systematic review in the last 1 year (n = 65)**	
Never before now	19 (29.2%)
Less than once per year	14 (21.5%)
1 to 4 times per year	21 (32.3%)
More than 5 times per year	11 (16.9%)
**Time spent reading provided evidence summaries (n = 65)**	
<12 hours	53 (81.6%)
>12 hours	6 (9.2%)
Did not read	6 (9.2%)
**Confidence in interpreting the term ‘randomisation’ (n = 65)** *	
Very confident	20 (30.8%)
Confident	33 (50.8%)
Not so confident	12 (18.5%)
**Description of a systematic review (n = 63)**	
Correct	39 (61.9%)
Incorrect	24 (38.1%)
**Interpretation of risk ratio (n = 64)**	
Correct	32 (50.0%)
Incorrect	32 (50.0%)
**Calculation of risk ratio of mortality comparing two treatments (n = 65)**	
Correct	16 (24.6%)
Incorrect	49 (75.4%)

† Denominator exclude missing data.

* Similar results were obtained for responses to questions ‘confidence in interpreting the term blinding’ and ‘confidence in interpreting the term selection bias’.

### Qualitative Findings

#### Views on the different formats for presenting systematic review evidence (see Box 1 for illustrative quotes)

The majority of participants interviewed found research information summarised in the form of narrative reports to be clearer, easy to read, easy to understand and containing ‘just the right amount of information’. Conversely, participants expressed considerable variability in views for systematic reviews and GRADE summary-of-findings tables: while some found the comprehensive and structured nature of information presentation in systematic reviews to be useful, a number expressed difficulties with extracting pertinent information. Some participants found GRADE summary tables to be good for ‘rapid consultation’; however, a number of participants found them difficult to understand as stand-alone summaries. For example, one participant expressed difficulties with distinguishing between the GRADE categories of quality of evidence. Of note, many participants reported lack of time and the volume of evidence as factors contributing to more complete engagement with the evidence.

#### Experiences with appraisal and use of research evidence (See Box 2 for illustrative quotes)

The majority of participants responded that they were not conversant with assessing the quality of scientific literature or evidence-based medicine terminology, such as PICO (Patient, Intervention, Comparison, Outcome). A number suggested that a short course on evidence-based medicine would be beneficial to support evidence-based guideline development.

## Discussion

### Interpretation of Results

The current study assessed the effectiveness and panelists’ views and experiences with different evidence summary formats. The quantitative evaluation showed that ‘graded-entry’ evidence summary formats which included narrative reviews highlighting local factors relevant for implementation, compared to full systematic reviews, were associated with higher composite scores for clarity and accessibility of information on critical outcomes. However, the proportion of correct answers to questions testing the ability to identify, access and reproduce key messages from the evidence summaries was not improved. This failure may reflect a true lack of effect of format on this outcome. Alternatively, it may be due to inadequate power to detect a smaller but useful effect or inadequate participant preparation in those with limited prior exposure to research evidence (Box 1 and table 5). Although not statistically significant, results of stratified analyses provide some support for the latter possibility as performance of policymakers and trainee paediatricians, arguably more familiar with interpreting evidence, was somewhat better. This interpretation of the trial findings, seen together with the qualitative data showing difficulties engaging with evidence among some panelists (Box 2), suggests that improving basic skills in evidence-based medicine may be important as we seek to engage wider participation in guideline development. Strategies to enhance panelists’ skills in evidence retrieval and interpretation may include: (1) involvement of panel members in the preparatory stages of guideline development (e.g. in the conduct of systematic reviews) [22], [23]; and (2) education of panel members on guideline development methods (e.g. on the GRADE system).

**Box 1. Panelists’ views on the research summary and presentation formats**

*[006]: ‘The mini-review [narrative report] I think was like one of the most useful things I came across, because I found that they had just the right amount of information…and also the Cochrane reviews I found were very good’*

*[010]: ‘…this particular one [table-of-key findings] was not very easy for me to understand…sometimes making a clear cut difference between especially the quality grades was not very easy…alone on itself I found it difficult…I needed to go back to the summaries, read a bit and come and compare…one needed to have some prior knowledge especially on how to interpret it…’*

*[012]: ‘The systematic reviews give more information on what was done in the research but you will need a lot of time to go through it. So if I were to be asked which one I preferred, I would pick the mini-review [narrative report] because it was very brief and easy to understand. It’s easy to understand and you don’t go and go until you get tired along the way’*

*[014]: ‘The Cochrane is good because it’s comprehensive and a very good summary of the work that’s available as well as what has been done. Its main disadvantage is that it takes more time to read through and digest. The mini-reviews [narrative reports] were the most useful because they were a condensed version, so they are easy to use even when you have limited time they will prove useful. The table-of-key findings is good for a rapid consultation but doesn’t give you a feel of how those conclusions were arrived at’*

*[016]: ‘The problem with the reviews is that most of them had limited data…But I was feeling that a lot more other data probably from observation studies could be available and would have been of more value. But at the end of the day you base most of your decisions on randomised controlled trials, but you should know a little more of what was done in the other studies’*

**Pre-workshop materials**

*[006]: ‘…like the people I was sitting with, most of them had not gone through all of the material, a few had gone through about half and a few had not looked at it at all’*

*[008]: ‘No, the reading of the materials appeared not to have been exhausting except you backed it up by making presentations…’*

*[010]: ‘Generally I think very few of the participants went through the materials before coming to the workshop. Personally I would say I didn’t spend adequate time that would have enabled me to understand the material better, for better participation during the workshop…’*

*[012]: ‘…and the volume of the document was another challenge. With the limited time people had and the volume of the document, people had very little time to go through them’*

**Box 2. Panelists’ experiences with appraisal and use of research evidence**

*[004]: ‘No, I can’t say I was comfortable, even when the PICO [Patient, Intervention, Comparison, Outcome] format was introduced I personally had problems understanding it…but now I understand it very properly and even the GRADE system, for me it really sunk well after the evidence week’*

*[011]: ‘Not at all, even being able to synthesize a research paper…it’s still a process that I would like to learn. For many of the participants I think a short course in that area would be useful and it would help a bit also in the voting’*

*[013]: ‘Most of us take it [research information] as it’s given to us; we don’t sieve it and see whether it’s of high or low quality…the general practitioners don’t really know the difference of [between] these studies’*

*[015]: ‘…I didn’t know there is low quality, moderate [quality] or even when I find in the books that it is low quality, I did not know actually what they based it on’*

Despite an absence of effect on our main outcome, ‘graded-entry’ summary formats and their components were reported to: improve clarity of information presentation, improve accessibility to key information, be more reader-friendly (both narrative reports and summary-of-findings tables) and were preferred by participants over systematic reviews. These formats are also likely to be more suited to the different backgrounds, interests and levels of expertise in multidisciplinary guideline panels. For example, those with limited training and skills in research synthesis (such as point of care clinicians) and busy policymakers may only need to scan the ‘front-end’ summaries for key results. On the other hand, those with expertise in research synthesis may prefer the detail available in narrative reports and systematic reviews to assess the reliability of the results before considering their applicability. These findings are further supported by the qualitative findings (Box 1). A preference for narrative reports may be due to: (1) their abbreviated and plain language nature; (2) incorporation of judgments on the quality of evidence for guideline relevant outcomes; and (3) inclusion of contextual information (e.g. local antimicrobial resistance patterns). A comparison of our findings with related studies is summarised in Box 3. However, it is clear that such ‘graded-entry’ formats are not by themselves an answer to the problem of improving the use of evidence in multidisciplinary guideline panels comprising important policy and provider groups - something that needs to be taken into account when structuring future approaches.

**Box 3. Research in context**

To compare current results with related studies we searched The Cochrane Library and PubMed (both from database inceptions up to March 2012) for articles examining ways of presenting evidence for use in healthcare decision-making (full search strategy available from the authors on request). The search identified six related studies: three systematic reviews [3], [24], [25], two randomised controlled trials [5], [26] and one qualitative study [15]. Only one [26] of the studies was conducted in a guideline development context. The systematic reviews noted that presentation of results of systematic reviews [3], [24] and health technology assessments [25] using ‘graded-entry’ formats (e.g. one page take home messages, a three-page executive summary and a 25-page report, 1∶3:25 format) rendered them more useful to healthcare managers and policymakers. Research syntheses using ‘graded-entry’ formats were however reported to be rare (identified in only 7% of 45 websites searched) [3].

In the first randomised controlled trial [5] participants attending an evidence-based practice workshop (N = 72; Norway) and a Cochrane Collaboration entities meeting (N = 33; UK) were allocated to receive a Cochrane review with or without a summary-of-findings table. Inclusion of a summary-of-findings table was found to significantly improve understanding and rapid retrieval of key findings. In the second randomised controlled trial [26], paediatricians and trainee paediatricians in private and public practice in Mexico (N = 216) were allocated to receive the same clinical recommendation presented using four different grading systems: NICE (National Institute for Health and Clinical Excellence), SIGN (Scottish Intercollegiate Guidelines Network), GRADE and Oxford CEBM (Centre for Evidence-Based Medicine). The primary outcome was mean change (before compared to after reading the guideline) in simulated clinician decision to use a therapy. The findings showed that clinician’s decision to use a therapy was influenced most by the GRADE system. However, no significant differences were found between systems in the clarity of presentation of care recommendation (defined as ‘ease of reading and undestanding’).

The qualitative interview (N = 13) study [15] explored health policymakers’ and managers’ experiences with short summaries of systematic reviews in five low- and middle-income countries, LMICs (Argentina, China, Colombia, South Africa, Uganda). The findings showed that participants preferred ‘graded-entry’ formats, particularly the section on the ‘relevance of the summaries for LMICs, which compensated for the lack of locally-relevant detail in the original review’.

Overall, observed differences in results across systematic reviews and primary studies examining impacts of evidence presentation formats, including our own, could be explained by differences in: study designs (randomised controlled trials versus user surveys), presentation formats (due to variations in layouts, contents, visual aids), types of participants (multidisciplinary versus unidisciplinary; variations in evidence based behaviors and skills); study contexts (evidence based medicine training versus policymaking environments); definitions of outcome measures (e.g. in García et al [26], unlike in other studies, clarity of presentation of recommendation was defined as ‘ease of reading and understanding’); and durations of exposure to the evidence summaries. These differences underscore the need for a unified system for evaluating impacts of evidence summary and presentation formats to enhance comparability and interpretation of results of accumulating work in this field.

### Implications for Development of Health Systems Guidance in Low- and Middle-Income Countries (LMICs)

We believe the results of our evaluation provide valuable inputs into methods for summarising and communicating evidence to those charged with formulating recommendations in LMICs. First, there is a need for wider sensitization to the principles of and tools for evidence-informed decision making. We also found our narrative reports, that adapted the GRADE system to synthesize and present evidence on treatment effects and on-the ground (contextual) factors and values that might influence intervention effectiveness, to be liked by busy decision makers. Such narrative reports could be made even more policy-relevant by inclusion of information on wider health systems perspectives such as: potential structural changes needed for effective implementation, equity, feasibility of scaling up and monitoring and evaluation issues. However, current findings could also inform future guideline development work in high-income countries.

### Strengths and Limitations

Combining a quantitative RCT design with qualitative interviews and using both in our interpretation of data improved our understanding of, and confidence in the study results. Our decision to study multiple tracer-topics rather than focus on a single guideline development topic may be considered a weakness or a strength. Perhaps if participants had only to read the literature provided for one topic, ‘graded-entry’ formats may have proved superior even on the primary outcome. However, in a low-income country setting like ours, it seems unlikely that countries will have the resources in the near future to engage specific panels for meetings for each guideline topic. Thus, we deliberately wished to examine a potentially efficient approach in which the panel considered multiple topics. Finally, although our randomization strategy (stratified by job-type/seniority) may have reduced possible variation in format quality and expertise (within the population enrolled) on the main findings, we cannot rule out the possible influence of these factors on the intended outcomes.

### Future Research

Although our findings suggest possible benefits of ‘graded-entry’ formats, evidence about the best formats for improving actual understanding and use of research findings in decision making processes (a key aim in knowledge translation) appears to be lacking. Thus, further studies are needed to learn more about the relative effectiveness of the various evidence summary formats that are available and how these may be combined with alternative approaches.

### Conclusions

‘Graded-entry’ formats were found to improve clarity and accessibility of research evidence, although this was not reflected in the number of correct responses to key clinical questions. Providing a ‘front-end’ summary of key information linked to locally relevant factors that support implementation, and the full systematic review, may help those developing guidelines access and contextualise research evidence.

## Supporting Information

Box S1 **Research synthesis products.**
(DOC)

Webappendix S1 **Front-end evidence summary.**
(PDF)

Webappendix S2 **Randomisation.**
(DOCX)

Webappendix S3 **Distribution of value and accessibility scores.**
(DOCX)

Webappendix S4 **Data handling and analysis.**
(DOCX)
