# Supplementary material for: Comparison of Alternative Evidence Summary and Presentation Formats in Clinical Guideline Development: A Mixed-Method Study
Source: PLoS One. 2013 Jan 25;8(1):e55067. doi: 10.1371/journal.pone.0055067 (PMC3555827; doi:10.1371/journal.pone.0055067)
Supplement: Box S1 — Research synthesis products. (DOC) [file pone.0055067.s001.doc]

**Box S1.** Examples of research synthesis (systematic review derived) products

| **Type of synthesis** | **Target audience** | **Description (Scope)** | **Source** |
| --- | --- | --- | --- |
| **Abstracts of Reviews of Effects** | Policymakers, clinicians | Quality-appraised summary of single non-Cochrane systematic reviews of effects of healthcare or health system interventions (delivery and organisation of health services) | Centre for Reviews and Dissemination (CRD)  <http://www.crd.york.ac.uk/CMS2Web/AboutDare.asp> |
| **Cochrane PICO** | Healthcare practitioners and decision-makers | Short summaries (about one page) of a clinical question addressed by one or more Cochrane reviews | Cochrane Editorial Unit  <http://editorial-unit.cochrane.org/cochrane-pico> |
| **Evidence Update** | Healthcare practitioners, policymakers | Two-page summaries of a Cochrane review of effects of healthcare or health system interventions relevant to people in low and middle-income countries (LMICs) | Effective Health Care Research Programme Consortium  <http://www.liv.ac.uk/evidence/evidence.htm> |
| **Plain language summaries of systematic reviews** | Policymakers, researchers, clinicians | Structured summaries of systematic reviews of effects of healthcare or health systems interventions in maternal and child health in LMICs  Summaries prepared using GRADE system | SUPPORT Collaboration  <http://www.support-collaboration.org/summaries.htm> |
| **Policy briefs** | Policymakers, researchers, civil societies | Summaries of policy-relevant reviews (healthcare, health systems, behaviour-change interventions) with a focus on LMICs  Summaries prepared using ‘graded entry’ formats (i.e., a list of key messages, an executive summary, and a full report) | SURE  [http://www.evipnet.org/local/SURE%20Website/home%20page.htm](http://www.evipnet.org/local/SURE Website/home page.htm)  EVIPNet  [http://www.evipnet.org/local/SURE%20Website/home%20page.htm](http://www.evipnet.org/local/SURE Website/home page.htm) |
| **Policy Liaison Initiative summaries** | Policymakers | |  | Summaries of policy-relevant Cochrane reviews of of effects of healthcare, health system, behaviour change and consumer-targeted interventions | | --- | --- | | Australasian Cochrane Centre  <http://www.cochrane.org.au/ebpnetwork/> |
| **Structured evidence summaries** | Healthcare professionals, and consumers | Summaries of multiple primary studies, systematic reviews, or evidence-based clinical guidelines of diagnostic, therapeutic and delivery of healthcare interventions | Joanna Briggs Institute (JBI)  <http://connect.jbiconnectplus.org/> |
| **Synopsies of reviews and single articles** | Clinicians | Structured abstracts of systematic reviews and original studies of diagnostic and therapeutic interventions | The American College of Physicians (ACP) Journal Club  <http://acpjc.acponline.org/shared/purpose_and_procedure.htm> |
| **2-page** [**summary statements**](http://www.health-evidence.ca/downloads/Summary statement template_Doc 9.pdf) | Healthcare practitioners, decision-makers, and managers | 2-page [summary statements](http://www.health-evidence.ca/downloads/Summary statement template_Doc 9.pdf) synthesizing results of systematic reviews of health promotion and public healthcare interventions | Health-evidence.ca  <http://www.health-evidence.ca/html/AboutUs> |

GRADE=Grading of Recommendations Assessment, Development and Evaluation; SUPPORT=Supporting Policy Relevant Reviews and Trials; SURE= Supporting the Use of Research Evidence; EVIPNet=Evidence-Informed Policy Network; PICO=Population (Patients, Problem), Interventions (Exposure), Comparison (Control), Outcomes
