# Supplementary material for: Comparison of Alternative Evidence Summary and Presentation Formats in Clinical Guideline Development: A Mixed-Method Study
Source: PLoS One. 2013 Jan 25;8(1):e55067. doi: 10.1371/journal.pone.0055067 (PMC3555827; doi:10.1371/journal.pone.0055067)
Supplement: Webappendix S1 — Front-end evidence summary. (PDF) [file pone.0055067.s002.pdf]

Child and Newborn Health Group

Kenya Medical Research Institute wellcome trust

Evidence summary: Kangaroo mother care for low birth weight infants

Clinical need

Kangaroo mother care (KMC), defined as continuous skin to skin between a mother and her newborn allowing frequent and exclusive breastfeeding, has been proposed as an alternative to conventional care (incubators, cots, etc) for low birth weight (LBW, less than 2500 g) babies. The evidence for the effectiveness and safety of KMC versus conventional care in LBW infants is considered in this summary.

Clinical questions, Quality of evidence‡ and Key findings

- **Population:** Low birth weight infants, less than 2500 g
- **Comparisons:** Kangaroo mother care versus conventional care
- **Outcomes:** Neonatal mortality, morbidity, breastfeeding status, costs and length of hospital stay

1. What is the evidence that KMC reduces the risk of mortality in LBW infants?
Key findings<br>• **Low quality evidence** suggests that KMC does not reduce the risk of death in ***stabilized*** LBW infants<br>• **Low quality evidence** suggests that KMC may reduce the risk of death in LBW infants if initiated very early in life ***before stabilization***
**2. What is the evidence that KMC reduces morbidity in LBW infants?**
Key findings<br>• **Low quality evidence** suggests that KMC reduces the risk of morbidity (mild / illnesses, nosocomial infections) in LBW infants.
**3. What is the evidence that KMC improves breastfeeding outcomes in LBW infants?**
Key findings<br>• **Low quality evidence** suggests that KMC increases the likelihood of exclusive breastfeeding at discharge in LBW infants

- **Moderate quality evidence** suggests that KMC increases the likelihood of exclusive breastfeeding at 41 weeks corrected age in LBW infants
- **Very low quality evidence** suggests that KMC may improve the chances of exclusive breastfeeding of LBW infants at the age of six months post birth

4. What is the evidence that KMC reduces the length of hospital stay of LBW infants?

Key findings

- **Low quality evidence** suggests that LBW babies on KMC stay hospitalized for a shorter duration compared to those on conventional care

5. What is the evidence for the cost-benefit of KMC compared to standard neonatal care?

Key findings

- **Very low quality evidence** suggests that the cost of care for babies on KMC is lower than the costs of standard care

‡ Quality of evidence is categorized as 'high', 'moderate', 'low' or 'very low'.

- **HIGH**: Further research is very unlikely to change our confidence in the estimate of effect.
- **MODERATE:** Further research is likely to have an important impact on our confidence in the estimate of effect and may change the estimate.
- **LOW:** Further research is very likely to have an important impact on our confidence in the estimate of effect and is likely to change the estimate.
- **VERY LOW**: We are very uncertain about the estimate.

Quality of Evidence and Summary of Findings

Question 1: What is the evidence that KMC reduces the mortality risk in LBW infants?

Intervention: Kangaroo mother care
Comparison: Conventional care
Bibliography: Worku et al[3]; Charpak et al[4]; Sloan et al[5]; Cattaneo et al[5]; Suman et al[9]

Quality assessment							Summary of findings		Importance
							Effect size (95% CI)	Quality (GRADE)	
No of studies	No of infants	Design	Limitations	Inconsistency	Indirectness	Imprecision			
**Mortality before stabilization (follow-up 4 to 6 days)**									
1	123	randomised controlled trial	serious†	no serious inconsistency	no serious indirectness	serious‡	RR 0.57 (0.33 to 1.0)	⊕⊕⊖⊖ **LOW**	CRITICAL
**Mortality after stabilization (follow-up 1 to 7 weeks)**									
4	1512	randomised controlled trials	serious††	no serious inconsistency	no serious indirectness	no serious imprecision	RR 0.70 (0.41 to 1.21)	⊕⊕⊕⊖ **MODERATE**	CRITICAL

† - significant number of recruited infants not randomised, blinding of investigators / data collectors unclear; ‡ small sample size, wide 95% confidence interval; †† blinding of investigators / data collectors unclear, potential for reporting bias (selective reporting of outcomes)

Question 2: What is the evidence that KMC reduces morbidity in LBW infants?

Intervention: Kangaroo mother care
Comparison: Conventional care
Bibliography: Charpak et al[4] Sloan et al[5]; Cattaneo et al[6]; Charpak et al[7]

Quality assessment							Summary of findings		Importance
							Effect size (95% CI)	Quality (GRADE)	
**No of studies**	**No of infants**	**Design**	**Limitations**	**Inconsistency**	**Indirectness**	**Imprecision**			
**Morbidity (risk of severe illness at discharge)**									
1	285	randomised controlled trial	serious†	no serious inconsistency	no serious indirectness	serious‡	RR 0.51 (0.28 to 0.94)	⊕⊕⊖⊖ **LOW**	CRITICAL
**Morbidity (risk of infectious episodes at 40 to 41 weeks corrected age; follow-up 3 to 10 weeks)**									
1	746	randomised controlled trial	serious¶	no serious inconsistency	no serious indirectness	serious‡	RR 1.06 (0.72 to 1.54)	⊕⊕⊖⊖ **LOW**	CRITICAL
**Morbidity (risk of nosocomial infections at 40 to 41 weeks corrected age; follow-up 3 to 10 weeks)**									
1	285	randomised controlled trial	serious¶	no serious inconsistency	no serious indirectness	serious‡	RR 0.47 (0.30 to 0.73)	⊕⊕⊖⊖ **LOW**	CRITICAL
**Morbidity (risk of severe illness at 6 months (follow-up 0-6 months)**									

1	275	randomised controlled trial	serious¶	no serious inconsistency	no serious indirectness	serious‡	RR 0.90 (0.30 to 0.66)	⊕⊕⊖⊖ **LOW**	CRITICAL
**Morbidity (risk of severe illness at 1 year corrected age; follow-up 0 to 12 months)**									
1	285	randomised controlled trial	serious††	no serious inconsistency	no serious indirectness	serious‡	RR 0.95 (0.06 to 15.09)	⊕⊕⊖⊖ **LOW**	CRITICAL

† - blinding of intervention to both investigators and data collectors unclear; ‡ - few number of events; ¶ - unclear concealment of intervention allocation, unblinded outcome assessment; †† - significant loss to follow-up

Question 3: What is the evidence that KMC improves breastfeeding outcomes in LBW infants?

Intervention: Kangaroo mother care
Comparison: Conventional care
Bibliography: Cattaneo et al[6]; Hake-Brooks et al[11]; Rojas et al[12]; Boo et al[13]

Quality assessment							Summary of findings		Importance
							Effect size (95% CI)	Quality (GRADE)	
No of studies	No of infants	Design	Limitations	Inconsistency	Indirectness	Imprecision			
**Breastfeeding (exclusively at discharge; follow-up 0 to 30 days)**									
4	537	randomised controlled trial	serious†	no serious inconsistency	serious‡	no serious imprecision	RR 1.33 (1.17 to 1.5)	⊕⊕⊖⊖ **LOW**	IMPORTANT
**Breastfeeding (exclusively at 40 to 41 weeks corrected age; follow-up 0 to 10 weeks)**									
1	746	randomised controlled trial	serious†	no serious inconsistency	no serious indirectness	no serious imprecision	RR 1.02 (0.87 to 1.21)	⊕⊕⊕⊖ **MODERATE**	IMPORTANT
**Breastfeeding (exclusively at 6 months of age; follow-up mean 6 months)**									
1	66	randomised controlled trial	serious¶	no serious inconsistency	serious‡	serious††	RR 2.51 (0.11 to 59.53)	⊕⊖⊖⊖ **VERY LOW**	IMPORTANT
**Breastfeeding (follow-up 0 to 12 months)**									

2	759	randomised controlled trial	serious†	no serious inconsistency	serious‡	serious††	RR 0.92 (0.69 to 1.23)	⊕⊖⊖⊖ **VERY LOW**	IMPORTANT

† - unclear concealment of allocation of interventions / blinding of outcome assessment; ‡ - one of the included studies conducted in a high income setting with a lactation consultant; ¶ - unclear concealment of allocation of interventions; †† - small number of events (<300)

Question 4: What is the evidence that KMC reduces the length of hospital stay of LBW infants?

Intervention: Kangaroo mother care
Comparison: Conventional care
Bibliography: Kadam et al[8]; Gathwala et al[10]; Boo et al[13]

Quality assessment							Summary of findings		Importance
							Effect size (95% CI)	Quality (GRADE)	
**No of studies**	**No of infants**	**Design**	**Limitations**	**Inconsistency**	**Indirectness**	**Imprecision**			
**Length of hospital stay (follow-up 0 to 40 days)**									
1	126	randomised controlled trial	serious†	no serious inconsistency	no serious indirectness	serious‡	13.5 days (KMC) versus 22.5 days (CMC)	⊕⊕⊖⊖ **LOW**	IMPORTANT
**Length of hospital stay (follow-up 0 to 2 months)**									
2	199	randomised controlled trial	serious†	serious¶	no serious indirectness	serious‡	KMC = shorter duration of hospital stay††	⊕⊖⊖⊖ **VERY LOW**	IMPORTANT

† - unclear concealment of allocation of interventions / blinding of outcome assessment /selective reporting of outcomes; ‡ - small number of enrolled participants; CMC - conventional method of care ¶ - age at randomisation into KMC group inconsistent across included studies; †† - Gathwala et al; (KMC, 3.56 days versus CMC, 6.8 days), Kadam et al (KMC, 8.5 days versus CMC, 9.3 days)

Question 4: What is the evidence that KMC reduces the length of hospital stay of LBW infants?

Intervention: Kangaroo mother care
Comparison: Conventional care
Bibliography: Sloan et al[5]; Cattaneo et al[6]

**Quality assessment**							**Summary of findings**		**Importance**
**No of studies**	**No of infants**	**Design**	**Limitations**	**Inconsistency**	**Indirectness**	**Imprecision**	**Effect size (95% CI)**	**Quality (GRADE)**	
**Cost of care (follow-up 0 to 6 months)**									
2	560	observational studies	very serious†	no serious inconsistency	serious‡	no serious imprecision	KMC = Lower costs of care¶	⊕⊖⊖⊖ **VERY LOW**	IMPORTANT

† - potential for selection / investigator bias in recruitment of participants / measurement of outcomes; ‡ - items costed unclear; ¶ - Sloan et al (KMC, US $101 versus CMC, US $130), Cattaneo et al (KMC, US $7,501 versus CMC, US $9,876)

Characteristics of the evidence

This evidence summary is based on a comprehensive search and critical appraisal (for methodological rigor and clinical practice applicability) of best currently available literature. The evidence in this summary comes from:

- One Cochrane review of randomised controlled trials (RCTs) (N=1,362 infants, 3 studies)[1]
- One overview of 2 systematic reviews and 7 RCTs[2]
- Ten RCTs (N=2,086 infants)[3-12]

References

1. Conde-Agudelo A, Belizán JM. *Kangaroo Mother Care to reduce morbidity and mortality in low birth weight infants.* Cochrane Database Syst Rev. 2003; (2).
2. Blackwell K, Adriano C. *What is the evidence of Kangaroo mother care of the* very low *birth weight infants*. International Child Health Review Collaboration (ICHRC). Available at: www.ichrc.org/pdf/kangaroo.pdf, Accessed 17th May 2010.
3. Worku, B, Kassie A. *Kangaroo Mother Care: A randomized controlled trial on effectiveness of early kangaroo mother care for the low birth weight infants in Addis Ababa, Ethiopia.* J Trop Ped, 2005: 51(2); 93-7
4. Charpak N, Ruiz-Pelaez JG, *et al*. *A randomized, controlled trial of kangaroo mother care: results of follow-up at 1 year of corrected age.* Pediatrics, 2001:108(5); 1072-1079.
5. Sloan NL, Camacho LW, Rochas EP*, et al*. *Kangaroo mother method: randomised controlled trial of an alternative method of care for stabilized low-birthweight infants.* Maternidad Isidro Ayora Study Team. Lancet, 1994: 344(8925); 782-785.
6. Cattaneo A, Davanzo R, Worku B, *et al*. *Kangaroo mother care for low birth weight infants: a randomised controlled trial in different settings.* Acta Paed, 1998: 87; 976-985.
7. Charpak N, Ruiz-Palaez JG, *et al. Kangaroo mother versus traditional care for newborn infants < 2000 grams: a randomised controlled trial.* Pediatrics, 1997:100(4):682-688.
8. Kadam S, Binoy S, Kanbur W, *et al*. *Feasibility of kangaroo mother care in Mumbai.* Indian J Ped, 2005:72; 35-38.
9. Suman RP, Udani R, Nanavati R. *Kangaroo mother care for low birth weight infants: a randomized controlled trial*. Indian Pediatr, 2008: 45; 17–23.
10. Gathwala G, Singh B, Balhara B. *KMC facilitates mother baby attachment in low birth weight* infants. Indian J Ped, 2008: 75; 43-47.
11. Hake-Brooks SJ, Anderson S. *Kangaroo mother care and breast-feeding of mother-preterm infant dyads 0-18 months: A randomized, controlled trial.* Neonatal Network, 2008: 27; 151-159.
12. Rojas MA, Kaplan M, Quevedo M, *et al*. *Somatic growth of preterm infants during skin-to-skin care versus traditional holding: A randomized, controlled trial.* Developmental and Behavioral Pediatrics, 2003: 24; 163–168.
