# Supplementary material for: Comparison of Alternative Evidence Summary and Presentation Formats in Clinical Guideline Development: A Mixed-Method Study
Source: PLoS One. 2013 Jan 25;8(1):e55067. doi: 10.1371/journal.pone.0055067 (PMC3555827; doi:10.1371/journal.pone.0055067)
Supplement: Webappendix S2 — Randomisation. (DOCX) [file pone.0055067.s003.docx]

**Web appendix 2. Randomisation**

| Evidence summaries in pack A, B and C formats were prepared for three ‘tracer-interventions’ relevant to neonatal care where new guidelines were being considered and for which systematic reviews had been recently published: feeding regimens in sick newborns (#1) [[12]](#_ENREF_16), hand hygiene for infection prevention (#2)[[13],](#_ENREF_17) and kangaroo care for low birth weight babies (#3)[[14].](#_ENREF_18)  We defined the following ‘evidence pack + tracer-intervention’ combination sets. Each participant received as their pre-reading material one of these three ‘evidence pack + tracer-intervention’ combination sets.   - A#1, B#2, C#3 - A#2, B#3, C#1 - A#3, B#1, C#2   With the three evidence packs, and three ‘tracer-interventions’ a complete experimental design would have resulted in six possible combinations. However, we used a simplified and partial experimental design for reasons of feasibility.  We expected experience with use of research evidence to vary across the stakeholder groups invited to the guideline development workshop. We thus grouped participants into the following 5 strata according to their roles: non-specialist health workers (nurses and clinical officers) (stratum 1); expert clinicians (paediatricians, neonatologists) (stratum 2); policymakers (representing Ministry of Medical Services, World Health Organisation (WHO), United Nations Children’s Fund (UNICEF)) (stratum 3); trainee paediatricians (stratum 4); and those with at least some experience of conducting systematic reviews (stratum 5).  By randomly allocating participants (in a 1:1:1 ratio within each of the 5 strata) to receive one of the three ‘evidence pack + tracer-intervention’ combination sets, we ensured: (1) that all participants received evidence on all three tracer-interventions; (2) that all participants were exposed to each of the three packaging formats; (3) that possible confounding of the relationship between evidence pack and the outcomes by tracer-intervention was reduced; (4) that subsequent interviewees would be able to reflect on the comparative value of each packaging format.  Figure 1 outlines the stratified randomisation process. One investigator (NO) generated the random allocation sequence (using a computer random number generator) and assigned participants to the different trial groups in sequential order. Participant recruitment was based on agreement to join the guideline development panel, attend its meeting and complete a self-administered questionnaire. We aimed to recruit a study population which represented those involved in guideline development and implementation in a low-income country. |
| --- |
