# Supplementary material for: Comparison of Alternative Evidence Summary and Presentation Formats in Clinical Guideline Development: A Mixed-Method Study
Source: PLoS One. 2013 Jan 25;8(1):e55067. doi: 10.1371/journal.pone.0055067 (PMC3555827; doi:10.1371/journal.pone.0055067)
Supplement: Webappendix S3 — Distribution of value and accessibility scores. (DOCX) [file pone.0055067.s004.docx]

**Webappendix S3.** Distribution of ‘value and accessibility’ scores
