# Supplementary material for: Comparison of Alternative Evidence Summary and Presentation Formats in Clinical Guideline Development: A Mixed-Method Study
Source: PLoS One. 2013 Jan 25;8(1):e55067. doi: 10.1371/journal.pone.0055067 (PMC3555827; doi:10.1371/journal.pone.0055067)
Supplement: Webappendix S4 — Data handling and analysis. (DOCX) [file pone.0055067.s005.docx]

**Webappendix S4. Data handling and analysis**

| Primary outcome   1. Data handling  - The primary outcome was understanding of key information (as measured by the proportion of correct responses to key evidence pack findings). - The 3-point responses (1=Correct, 2=Not clear, 3=Incorrect) were recoded into a binary response variable (0=Incorrect, 1=Correct). - Each of the three tracer-interventions contributed two questions in the binary response variable (n=6 questions). - All participants received each of the three tracer-interventions; hence, each participant contributed six responses in the overall binary response variable.  1. Method of analysis  - The odds of correct responses for pack B and C compared to the odds for pack A (assumed baseline pack) were estimated by calculating odds ratios (ORs) and 95% confidence interval (CIs) using logistic regression. - To assess whether the effects of packs were modified by the type of strata, logistic regression model with type of pack and strata as an interaction term (pack*strata) was performed. Further tests of interactions were performed using likelihood ratio tests. Where evidence of interaction was found, stratum-specific ORs and 95% CIs were calculated.   Secondary outcome   1. Data handling  - The secondary outcome measure was mean ‘value and accessibility’ score. Value measure was participant self-report of perceived clarity of presentation of key information (1=Agree, 2=Not clear, 3=Disagree). Accessibility measures were self-reported ease of locating information on critical / important outcomes and judgments about the quality of evidence for critical / important outcomes; participants self-rated their responses on a 5-point scale. - For both of the measures of access, we recoded the 5-point Likert scores (1=Strongly, 2= Disagree, 3=Neither agree nor disagree, 4=Strongly agree, 5=Agree) into 3-point Likert scores (1=Strongly disagree/Disagree, 2=Neither agree nor disagree, 3=Agree/Strongly agree). - The 3-point ‘value and accessibility’ scores (responses) were further recoded to assume a common direction – meaning that scores ranged from 1 to 3 with the highest score interpreted as the most ‘valuable / accessible’. - Each of the three tracer-interventions contributed three questions to the 3-point Likert ‘value and accessibility’ score. Hence, each participant contributed 9 responses in the overall ‘value and accessibility’ score.  1. Method of analysis  - A mean ‘value and accessibility’ score for each of the three tracer-interventions was derived by summing up scores of responses (range, 3 to 9) and dividing by three. Thus, each individual contributed three mean scores to the analysis. - The ‘value and accessibility’ scores were not normally distributed. (See histogram, figure 2). Methods that correct for non-normal outcome distributions were therefore applied (i.e. resampling by ‘bootstrapping’[24]) to calculate estimates of pack effects. - The mean ‘value and accessibility’ scores of pack B and pack C compared to the mean scores of pack A were estimated using linear regression. - To confirm the results of linear regression analysis, an alternative approach to the analysis was undertaken: the odds of a one point increase in the ‘clarity and accessibility’ scores of pack B and C compared to the odds of pack A were estimated using ordinal logistic regression models. Further tests of interactions between pack and strata were performed using likelihood ratio tests. Where evidence of interaction was found, stratum-specific ORs and 95% CIs were calculated.   All analyses were done with STATA (version 11.0). |
| --- |
